# Metabolomic analysis of follicular fluid from women with Hashimoto thyroiditis

**DOI:** 10.1038/s41598-023-39514-7

**Published:** 2023-08-02

**Authors:** Diana Caroline da Silva Bastos, Maria Izabel Chiamolera, Renata Elen Silva, Maria Do Carmo Borges de Souza, Roberto Azevedo Antunes, Marcelo Marinho Souza, Ana Cristina Allemand Mancebo, Patrícia Cristina Fernandes Arêas, Fernando M. Reis, Edson Guimarães Lo Turco, Flavia Fonseca Bloise, Tania M. Ortiga-Carvalho

**Affiliations:** 1 Laboratório de Endocrinologia Translacional, Instituto de Biofísica Carlos Chagas Filho, Universidade Federal do Rio de Janeiro, Rio de Janeiro, Brasil; 2 Laboratório de Endocrinologia Molecular e Translacional, Escola Paulista de Medicina, Universidade Federal de São Paulo, São Paulo, Brasil; 3 Fertipraxis Centro de Reproducao Humana, Rio de Janeiro, Brasil; 4 Maternidade Escola, Universidade Federal do Rio de Janeiro, Rio de Janeiro, Brasil; 5 Departamento de Ginecologia e Obstetrícia, Universidade Federal de Minas Gerais, Belo Horizonte, Brasil; 6 Ion Medicine, São Paulo, Brasil; 7 Departamento de Cirurgia, Disciplina de Urologia, Setor de Reprodução Assistida Universidade Federal de São Paulo, Universidade Federal de São Paulo, São Paulo, Brasil

**Keywords:** Reproductive biology, Endocrine system and metabolic diseases, Thyroid gland

## Abstract

Hashimoto thyroiditis is an autoimmune disease characterized by hypothyroidism and a high level of anti-thyroid autoantibodies. It has shown to negatively impact female fertility; however, the mechanisms are unclear. Ovarian follicular fluid appears to be the key to understanding how Hashimoto thyroiditis affecst fertility. Thus, we aimed to evaluated the metabolic profile of follicular fluid and antithyroid autoantibody levels in the context of Hashimoto thyroiditis. We collected follicular fluid from 61 patients, namely 38 women with thyroid autoantibody positivity and 23 women as negative controls, undergoing in vitro fertilization treatment. Follicular fluid samples were analyzed using metabolomics, and thyroid autoantibodies were measured. Fifteen metabolites with higher concentrations in the follicular fluid samples from Hashimoto thyroiditis were identified, comprising five possible affected pathways: the glycerophospholipid, arachidonic acid, linoleic acid, alpha-linolenic acid, and sphingolipid metabolism pathways. These pathways are known to regulate ovarian functions. In addition, antithyroglobulin antibody concentrations in both serum and follicular fluid were more than tenfold higher in women with Hashimoto thyroiditis than in controls. Our data showed that the metabolic profile of follicular fluid is altered in women with Hashimoto thyroiditis, suggesting a potential mechanistic explanation for the association of this disease with female infertility.

## Introduction

Women with Hashimoto thyroiditis (HT) suffer more from miscarriage, recurrent pregnancy loss, decreased fertilization rates, and reduced numbers of good-quality embryos than euthyroid women^1–5^. HT is the most common autoimmune disease in reproductive age women^6^. The two autoantibodies associated with autoimmune destruction of the thyroid gland and with the clinical condition of hypothyroidism are the antithyroid peroxidase antibody (TPOAb) and the anti-thyroglobulin antibody (TGAb)^7^. Nonetheless, treatment of HT with thyroid hormone replacement does not seem to have a protective effect on fertility outcomes. HT patients with controlled thyroid hormone levels still present a low fertility rate^8^. Thus, the impact of HT on infertility cannot be associated only with hypothyroidism.

Follicular fluid (FF) and autoantibodies seem to play a central role in the relationship between HT and female infertility. The first study to propose this idea showed the presence of antibodies in FF, with higher levels in women with HT^1^. Subsequently, other studies investigated the presence of antibodies in FF and women’s fertility outcomes^4,9^. In addition to the increased levels of autoantibodies in FF, from women with HT, these women had lower oocyte fertilization rates, fewer grade A embryos, lower pregnancy rates and an increased risk of early miscarriage compared to euthyroid women^1,4^.

FF originates from the union of products released by granulosa and theca cells with blood exudate^10^. The composition of FF is different from that of serum^11^. FF contains metabolites that accumulate in the oocyte, promoting its maturation. FF is composed of amino acids, lipids, nucleotides, and essential factors for oocyte competence, such as hormones, cytokines, growth factors, proteins, and metabolites^12,13^.

Although some research has shown high concentrations of thyroid autoantibodies in FF from women with HT, it is still unknown whether these factors could explain how the disease affects fertility. Regarding protein expression, 49 proteins have been found to be differentially expressed in FF from HT patients compared to FF from control patients^14^. This finding suggests that HT can alter the composition of FF.

To confirm the possible impact of the disease on FF composition, it is necessary to evaluate the FF metabolic profile, since the metabolic composition of FF is important for oocyte development^12^. Thus, the change in the metabolic profile of FF may be the mechanism by which HT affects female fertility.

Metabolomic analysis of FF has already been performed to assess changes in ovarian follicles^15–17^. In addition, other studies have used the technique to further investigate the pathophysiology of HT in other samples, such as blood, urine, and thyroid biopsy samples^18–22^. However, applying metabolomics to understand the link between HT and changes in ovarian follicles, particularly changes in FF, is still an unexplored area.

The aim of this study was to investigate the impact of HT on FF by examining the levels of autoantibodies in and the metabolic profile. Understanding the mechanism by which HT can affect female fertility should provide valuable insight into the complex pathophysiology of the disease and a possible starting point for developing therapies targeted toward the associated subfertility.

## Results

### Laboratory and clinical information

**Figure 1:**
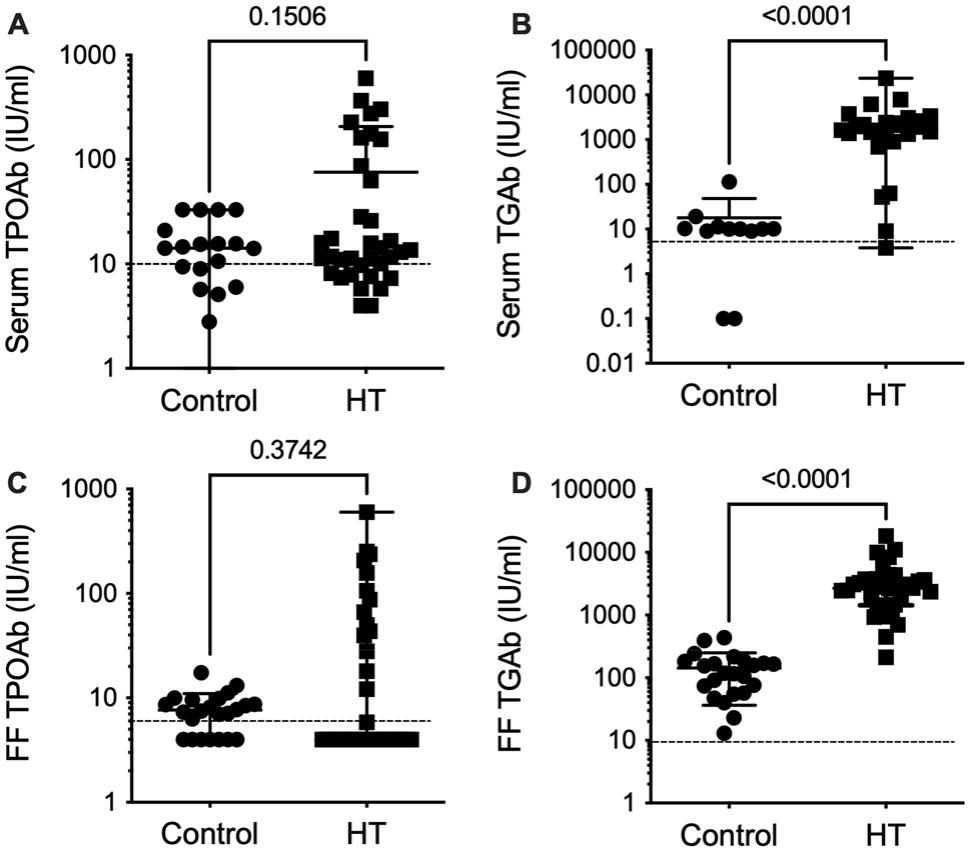
Anti-thyroid antibody levels in follicular fluid and serum samples from patients undergoing in vitro fertilization. (**A**) Serum anti-thyroid peroxidase (TPOAb), (**B**) Serum anti-thyroglobulin (TGAb), (C) Follicular fluid TPOAb, (D) Follicular fluid TGAb levels (shown for each group), Control (circles) and Hashimoto thyroiditis (HT squares). Data are expressed as the medians (quartiles). P values refer to the 2-sided Mann‒Whitney U test. The dotted line represents the limit of detection of each assay. Control n = 23. Hashimoto n = 38.

The participants with and without HT had similar concentrations of TSH, FT4, serum TPOAb and FF TPOAb (Table 1 and Fig. 1). However, serum and FF TGAb levels were dramatically increased in the patients with HT (*p* < 0.001, Fig. 1).

**Table 1:** Clinical and laboratory information of the control group and the Hashimoto group.

	Control	Hashimoto	*P* value
Age (years)	36 (35–39)	36 (32–38)	0.423
BMI (kg/m^2^)	21 (20–26)	23 (22–26)	0.183
Duration of infertility (months)	19 (12–36)	9 (5–14)	0.011
Previous miscarriages	3/16 (19%)	10/33 (30%)	0.502
Serum TSH (μIU/ml)	2.18 (1.46–3.64)	1.79 (2.29–2.99)	0.882
Serum FT4 (ng/dl)	1.20 (1.05–1.32)	1.10 (1.00–1.45)	0.660

Data are expressed as the medians (quartiles) or frequencies (percentages). P values refer to the 2-sided Mann‒Whitney U test. BMI: body mass index; TSH: thyroid-stimulating hormone; FT4: free thyroxine; Control n = 23. Hashimoto n = 38.

Even though only the TGAb concentrations showed significant differences between the groups (Fig. 1B,D), the concentrations of TPOAb were more dispersed and skewed in the HT group (Fig. 1A,C).

### In vitro fertilization parameters

After clinical evaluation, we investigated intermediary IVF outcomes between groups (Table 2). The parameters used in the study were the number of oocytes, proportion of metaphase II oocytes, fertilization rate and number of embryos. In the analyzed outcomes, we found no significant difference between the control and HT groups.

**Table 2:** In vitro fertilization outcomes in the two groups.

	Control	Hashimoto	*P* value
Number of oocytes	6 (4–15)	8 (3–13)	0.929
Proportion of MII oocytes (%)	80 (69–100)	81 (65–100)	0.559
Fertilization rate (%)	96 (79–100)	80 (57–100)	0.095#
Number of embryos	3 (3–6)	4 (1–7)	0.746

Data are expressed as the medians (quartiles). P values refer to the Mann‒Whitney U test. MII: metaphase II. Control n = 23. Hashimoto n = 38 except; # Control n = 18 and Hashimoto n = 34 due to cryopreservation of the oocytes from 8 patients.

### Metabolomics of follicular fluid

**Figure 2:**
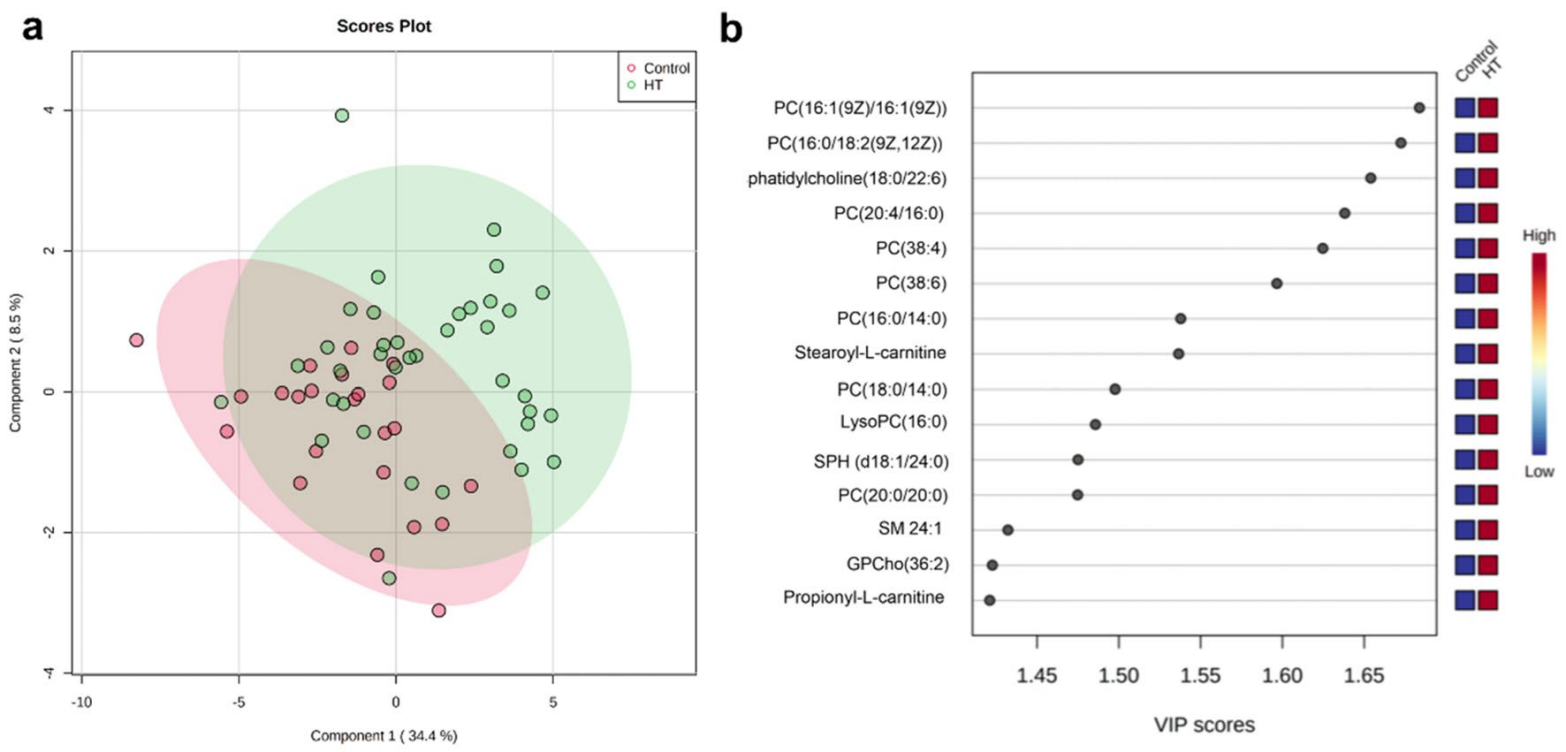
Metabolomic analysis of follicular fluid in control and Hashimoto's thyroiditis patients. (**a**) Two-dimensional score plot by PLS-DA of follicular fluid samples from the control group (red) and Hashimoto group (HT-green) of patients undergoing in vitro fertilization treatment. Control n = 23. Hashimoto n = 38 (**b**) Identification of important metabolite markers among the studied groups. The important features were identified by PLS-DA. The color boxes on the right indicate the relative concentrations of the corresponding metabolite in each group. The red-coloured box located below the group indicates that there is a high concentration of the metabolite in this group, while the blue-colored boxes represent low concentrations. Image generated by GraphPad Prism and MetaboAnalyst software. HT: Hashimoto’s group, PC/GPCho: phosphatidylcholine, SPH/SM: sphingomyelin, LysoPC: lysophosphatidylcholine. Control n = 23. Hashimoto n = 38.

In the FF metabolomic analysis, we detected and measured the concentrations of 83 metabolites. In the PLS-DA, it was possible to observe the different metabolic profiles in the FF of the two groups (Fig. 2a). Additionally, we found three metabolites with significant alterations in concentrations between the HT and euthyroid groups. The volcano plot depicts these significant alterations (Table 3). The complete dataset of metabolite identification, names, and concentrations is available at the Mendeley Data repository (https://doi.org/10.17632/nm5gzmdsjh.1).

**Table 3:** Volcano plot illustrating significant changes in FF concentrations of metabolites between patients with Hashimoto thyroiditis and controls.

Cmpds	FC	p value	Mean Control	SD Control	Mean HT	SD HT
PC (38:4)	0.48065	0.001059	5527.3	2701.3	11,499.7	7541.9
PC (20:0/20:0)	0.46384	0.004789	3960.1	1999.4	8537.7	7125.3
Adenosine	0.24489	0.068223	1.0	0.1	4.4	19.1

Cmpds: compounds; FC: fold change; SD: standard deviation; HT: Hashimoto thyroiditis.

The analysis of metabolites present in FF showed differences in 15 metabolites between the HT and euthyroid patients (Fig. 2b).

**Figure 3:**
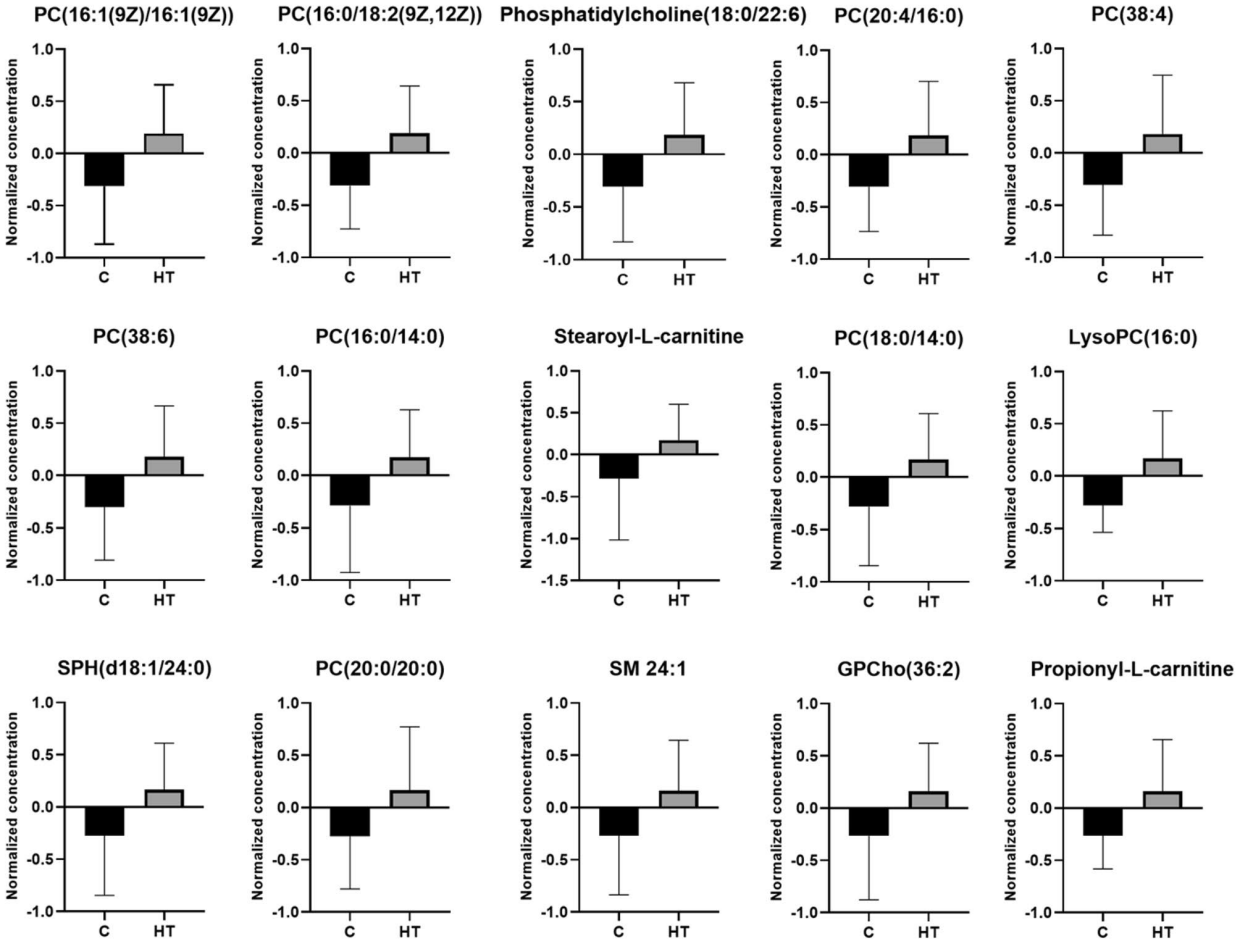
Normalized concentrations of 15 metabolic markers in the follicular fluid of patients. The red color represents the control group and the green color represents the Hashimoto group. Data are expressed as the mean and standard deviation. Hashimoto group, PC/GPCho: phosphatidylcholine, SPH/SM: sphingomyelin, LysoPC: lysophosphatidylcholine. Control n = 23. Hashimoto n = 38.

All 15 metabolites were found to have higher concentrations in the FF of HT patients. Among the metabolites found to be altered, there were ten phosphatidylcholines, two acylcarnitines, two sphingolipids, and one lysophosphatidylcholine. Quantitative analysis confirmed our data (Fig. 3).

### Pathway analysis

**Figure 4:**
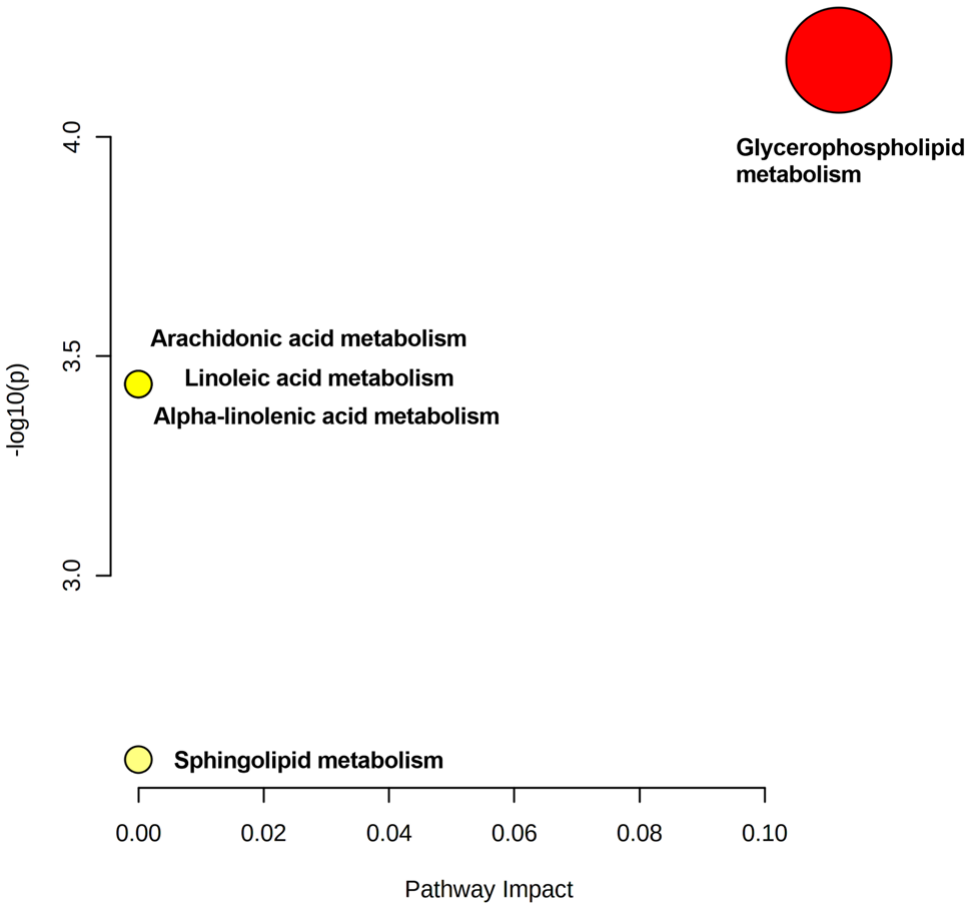
Pathway analysis summary showing altered follicular fluid metabolism of patients with Hashimoto's thyroiditis. The pathway impact value (x-axis) corresponds to the circle size, while the adjusted statistical significance (log of the P value, y-axis) corresponds to the circle colour (red>yellow). The circles representing arachidonic acid, linoleic acid and alpha-linoleic acid metabolism are superimposed. Image generated by the programs GraphPad Prism and MetaboAnalyst.

We searched for possible affected pathways based on the identified metabolites and their respective concentrations. We detected five altered pathways: the glycerophospholipid, arachidonic acid, linoleic acid, alpha-linolenic acid, and sphingolipid metabolism pathways (Fig. 4, Table 4).

**Table 4:** Pathway analysis results based on metabolic markers and their concentrations found in follicular fluid.

	Total Cmpds	Hits	Raw p	-log10 (p)	Holm adjusted	FDR	Impact
Glycerophospholipid Metabolism	36	2	6.68E-05	4.1751	0.000334	0.000334	0.11182
Arachidonic Acid Metabolism	36	1	0.000366	3.4363	0.001465	0.000458	0
Linoleic Acid Metabolism	5	1	0.000366	3.4363	0.001465	0.000458	0
Alpha-Linolenic Acid Metabolism	13	1	0.000366	3.4363	0.001465	0.000458	0
Sphingolipid Metabolism	21	1	0.00263	2.58	0.00263	0.00263	0

Cmpds: compounds; FDR: false discovery rate.

Among these pathways, the glycerophospholipid metabolism pathway exhibited the highest presence of identified metabolites, with two metabolites, phosphatidylcholine (PC) and lysophosphatidylcholine (LysoPC), being prominently present. The identification of the arachidonic acid, linoleic acid, and alpha-linolenic acid metabolism pathways can be attributed to the presence of the phosphatidylcholine metabolite. Additionally, the sphingolipid metabolism pathway was identified based on the presence of the sphingomyelin metabolite (SPH/SM).

## Discussion

The mechanism through which HT affects female fertility is still not known. However, our findings contribute to explaining this connection by showing important differences in the metabolites present in the FF of euthyroid HT patients compared to euthyroid non-HT controls for the first time.

The results of blood measurements of thyroid-related hormones and autoantibodies demonstrate that patients may have normal serum TSH, FT4, and TPOAb levels and still present with the disease, with high TGAb levels. This finding reinforces the importance of measuring thyroid autoantibodies in infertile women, since 5–10% of them may have HT^23^. Although the measurement of TPOAb is the most commonly used test for HT diagnosis^24^, our data indicate that serum TGAb levels may be a better marker of the disease. This result conflicts with the current understanding that TPOAb alone would be sufficient for investigating thyroid autoimmunity^25^. Although limited to women undergoing IVF, this finding is of clinical relevance since many studies and diagnoses focus only on TPOAb as a diagnostic marker for the disease.

Studies have presented results that stimulate discussion about the clinical relevance of the TGAb test^26–28^. A study involving pregnant patients obtained data that contradicted the importance of TGAb testing. The work showed that there are TPOAb-negative and TGAb-positive patients with normal thyroid function who may need treatment and recommended that TGAb be measured in TPOAb-negative pregnant patients^27^. Another group showed that pregnant patients with TPOAb positivity and patients with only TGAb positivity had different outcomes for their children^28^. Furthermore, in mice immunized with Tg and presenting high levels of Tg antibodies without thyroid dysfunction, a higher incidence of fetal resorption and reduced placental and embryo weights were observed^26^.

We found no difference in the evaluated IVF results in relation to the disease. Our findings contrast with previous research showing an increased miscarriage rate and a decreased fertilization rate in women with HT^1–5^; however, our results are consistent with other studies suggesting that HT does not affect IVF results^2,29–31^. A recent systematic review with meta-analysis suggested that studies evaluating different antibodies (TPOAb and/or TGAb) and different cutoff values may reach conflicting conclusions about the impact of autoimmune thyroiditis on IVF outcomes^31^. Therefore, we believe that our findings alone do not exclude a possible relationship between HT and IVF results.

The FF of HT patients had 15 metabolites with increased concentrations compared to the FF of controls. Our findings included an increase in phosphatidylcholine, acylcarnitine, lysophosphatidylcholine, and sphingolipids. Metabolomics has already been used to investigate HT in blood, urine, and thyroid biopsy samples^18–22^, but the metabolites found to be altered in these samples were different from those identified in ovarian FF in the present study. However, one of the metabolites that we found to be high in the FF of HT patients, PC (18:0/22:6), was reported to be increased in the blood of hyperthyroid patients^21^. Therefore, this is the second study that relates PC (18:0/22:6) with thyroid pathology, and further studies are needed to investigate the possible relationship.

The larger metabolite group in the FF of HT patients was phosphatidylcholine. We identified ten phosphatidylcholines: PC(16:1(9Z)/16:1(9Z), PC(16:0/18:2(9Z,12Z), phosphatidylcholine(18:0/22:6), PC(20:4/16:0), PC(38:4), PC(38:6), PC(16:0/14:0), PC (18:0/14:0), PC(20:0/20:0), and GPCho(36:2). Phosphatidylcholine is the lipid subclass that is most abundant in cell membranes. In porcine FF, phosphatidylcholine was found in higher concentrations in large antral follicles than in smaller antral follicles^32^. These data indicate that the synthesis of these metabolites may be involved in follicle growth and oocyte maturation. Furthermore, increased levels of phosphatidylcholine were found in serum and FF samples from patients with ovarian endometriosis^33,34^. Therefore, phosphatidylcholine appears to play an important role in ovarian physiology.

One of the metabolites we found to be differentially concentrated in FF from HT patients was a lysophosphatidylcholine, LysoPC(16:0). Lysophosphatidylcholine is secreted by cumulus cells^35^ and can induce an acrosome reaction in human sperm^36^. The acrosome reaction is necessary for sperm to be able to fertilize an oocyte. The use of a high concentration of this metabolite was able to induce an acrosome reaction in human sperm within 15 min. However, this same experiment also identified that sperm had a rapid loss of motility and a drop in viability^36^. The increased concentration of lysophosphatidylcholine in FF, which is released along with the oocyte at the time of ovulation, can decrease sperm motility and viability during fertilization in vivo. If the increase in FF lipid concentration can have adverse effects on sperm^36^, this could explain the difficulty that patients with HT experience in becoming pregnant naturally and why our study did not identify such a gap in pregnancy rates following IVF treatment. In IVF, the oocytes are removed from the FF and kept in culture medium, so the composition of the FF does not affect the sperm. Further experimental studies in suitable animal models are needed to directly test the hypothesis that increasing the concentration of lysophosphatidylcholine in FF can hinder in vivo fertilization.

We found an increase in acylcarnitines, propionyl-L-carnitine, and stearoyl-L-carnitine in the FF of women with HT. Acylcarnitine is responsible for carrying out the beta-oxidation of fatty acids. This process is one of the most important pathways for producing metabolic energy^37^. Acylcarnitine supplementation is protective against mouse oocyte cytoskeletal damage and embryonic apoptosis induced by incubation in the peritoneal fluid of patients with endometriosis^38^. Furthermore, this supplementation in mice increases beta-oxidation and improves the rate of fertilization and the development of embryos^39^. In addition to findings in animal models, acylcarnitine has also been shown to be important in studies involving humans. A reduction in acylcarnitine levels has been previously noted in serum and FF samples from patients who had more than 9 oocytes and more than 6 embryos^40^. The study suggests that in patients with better reproductive potential, this pathway is upregulated and, therefore, there is high consumption of acylcarnitine, with its concentrations found at low levels^40^. Based on this work, the increase in acylcarnitine levels in the FF of women with HT in the present study can be related to low consumption of this metabolite, providing a further potential mechanism for the subfertility associated with HT, which can be overcome by IVF. More work is needed to investigate the relationship of acylcarnitine with reproductive potential and disease.

Sphingomyelins are a type of sphingolipid found in animal cell membranes. Here, we found an association between HT and increased FF levels of sphingomyelins SM 24:1 and SPH (d18:1/24:0), which are important components of the sphingomyelin pathway. In addition, sphingolipids have been linked to ovarian cancer and ovarian endometriosis^33,34,41^. An increase in this metabolite in the blood has been associated with a risk of developing ovarian cancer 3 to 23 years before diagnosis^41^.

The metabolic changes found in FF suggest changes in five pathways, namely, the glycerophospholipid, arachidonic acid, linoleic acid, alpha-linoleic acid, and sphingolipid pathways. The first involves phosphatidylcholine and lysophosphatidylcholine. This was previously reported to be dysregulated in patients with ovarian endometriosis^42^. Arachidonic acid and linoleic acid derivatives play relevant roles that can affect fertility and pregnancy. Some of its derivatives may be predictive markers of pregnancy complications such as gestational diabetes mellitus or preeclampsia^43^.

In the sphingolipid pathway, there are metabolites related to the apoptosis process, such as ceramide and sphingosine, and metabolites related to cell survival in response to apoptotic stimuli, such as sphingosine-1P^44^. Thyroid hormones, mainly T3, induce the survival of human granulosa cells, both in the preovulatory stage and in the luteinized phase^45,46^. Hypothyroidism, which occurs in HT when untreated, could potentially lead to the apoptosis of granulosa cells due to the increase in the sphingolipid pathway and the decrease in T3.

All our findings involving the metabolomics of FF seem to be directly involved in ovarian physiology and female fertility, although we did not find any significant impact on the patients’ IVF results. This suggests that HT may affect follicle development and oocyte fertilization to an extent that can be bypassed by IVF through ovarian stimulation and in vitro procedures. However, this study has some limitations. We first used metabolomics as an exploratory strategy to identify potential pathways in ovarian FF affected by HT, and our results should be interpreted as hypothesis-generating rather than conclusive demonstration of such mechanisms. The relationship of FF metabolites and their pathways with ovarian physiology and natural fertility should be further studied in animal models due to ethical constraints in obtaining this biological fluid from women who are trying to conceive spontaneously. In the context of IVF, our findings may direct hypothesis-driven studies to clarify whether the presence of HT has any implication for gamete and embryo development beyond the fertilization rate and gross embryo morphology.

In this study, we observed that the serum TGAb level may be a more consistent biomarker of HT in women preparing for IVF than the serum TPOAb level, which is the standard HT marker used in clinical practice. For the first time, metabolomics was used to assess the metabolic profile present in the FF of women with autoimmune disease, and the data showed that the metabolic profile of FF is altered in HT patients. Furthermore, these findings may explain how the presence of autoantibodies in FF can impact ovarian follicles and female fertility.

## Materials and methods

### IVF patients

The study had a prospective design. All patients eligible for IVF treatment at Fertipraxis, a reproductive medicine center located in the city of Rio de Janeiro, from 2019 to 2020 were considered potential participants. The exclusion criteria were IVF cycle cancellation or patients who had no thyroid function information. A total of 61 women undergoing IVF were divided into two groups according to the blood levels of thyroid autoantibodies (TPOAb levels > 34 IU/mL and/or TGAb levels > 115 IU/mL) or a previous diagnosis of HT. Thus, 38 euthyroid women had thyroid autoantibody positivity and 23 were negative controls. After the selection of the patients for each group, all measurements were blinded to the researchers until final analysis. Although we selected a total of 61 patients, some parameters were evaluated in fewer patients according to the information that was available. TGAb blood tests were available for 38 patients (26 from the HT group and 12 from the control group), while TSH and free T4 were assessed in 37 participants (14 in the HT group and 23 in the control group). Regarding the fertilization rate, 34 patients in the HT group and 18 in the control group were evaluated because eight patients had all oocytes cryopreserved. All subjects signed an informed consent form during their first medical evaluation. None of the patients were categorized by ethnicity due to the mixed genetic heterogeneity of the Brazilian population. This research was approved by the Research Ethics Committee on Research at the Maternidade Escola, Universidade Federal do Rio de Janeiro and registered on Plataforma Brasil under number 02213812.4.0000.5275. Consent was obtained from all participants and all experiments were performed in accordance with relevant named guidelines and regulations.

### FF thyroid autoantibody measurements

FF was collected during follicular aspiration and stored at − 20 °C for future analyses. The fluid was used to measure autoantibody levels and to perform metabolomic tests. To investigate the concentrations of TPOAb in FF, we used the Elecsys kit competition immunoassay method (ROCHE, Mannheim, Germany) following the manufacturer’s instructions. The limit of detection was 5 UI/mL. The levels of TGAb in FF were measured by an “*in house*” assay, which has been previously described and validated^47^. The limit of detection was 10 UI/mL.

### FF metabolomic analysis

Metabolite analysis of the patients’ FF samples was performed using the liquid chromatography method coupled to an electrospray ionization mass spectrometer (LC–ESI–MS). The FF data obtained by multiple reaction monitoring (MRM) and deuterated standards inside each sample were analyzed using predetermined methods stored in the equipment library. The resulting data, including the concentration of each identified metabolite in the FF and its normalization, are accessible at the Mendeley Data repository under the number: https://doi.org/10.17632/nm5gzmdsjh.1.

The LC‒MS analyses were performed on a Nexera CL HPLC System (Shimadzu) consisting of a degasser unit, two unitary pumps, an autosampler, a column oven and a controller module. HPLC was coupled to a triple-quadrupole LCMS-8060 CL mass spectrometer (Shimadzu). The injection volume in all cases was 5 µL, and ionization was performed via electrospray ionization (ESI) in positive and negative modes, depending on the analyte. The analyses were performed using a targeted approach, and the ions were assessed by multiple reaction monitoring (MRM).

The mobile phases were prepared with acetonitrile (ACN), water and formic acid (Merck) in different proportions, all of which were LC‒MS grade. The results were acquired using three different methods to evaluate the following diverse classes of the molecules of interest: (I) primary metabolites (acquired from Shimadzu); (II) lipid mediators (acquired from Shimadzu); and (III) lipids and carcinogens (in house development). The methods are detailed in the next paragraphs.

The mobile phase for method (I) – primary metabolites – was composed of acid 0.1% (v/v) (A) and ACN + formic acid 0.1% (v/v) (B). The analyses were carried out using a PFFP (3 µm, 150 mm × 2.1 mm, Supelco) column at 40 °C. The gradient of B, at a flow of 0.25 mL/min, was as follows: time 0, 0%; 1 min, 0%; 2 min, 25%; 11 min, 35%; 15 min, 95%; 20 min, 95%; 20.1 min, 0%; and 25 min, 0%.

Method (II)—lipid mediators—was performed using a Kinetex C8 (2.6 µm, 150 mm × 2.1 mm, Phenomenex) column at 40 °C. The mobile phase was acid 0.1% (v/v) (A) and ACN (v/v) (B), the flow was 0.40 mL/min, and the gradient for B was as follows: time 0, 10%; 5 min, 25%; 10 min, 35%; 20 min, 75%; 20.1 min, 95%; 25 min, 95%; 25.1 min, 0%; and 28 min, 0%.

The column and mobile phase for method (III)—lipids and carnitines—was the same as for method (II). The column oven was set at 70 °C and the initial flow was 0.45 mL/min. The gradient for B, totalling 28 min of analysis, was as follows: time 0, 5%; 8 min, 60%; 20 min, 80%; 21 min, 98%; 26 min, 98%; 26,1 min, 5%; and 28 min, 5%. A flow gradient was also used for this method: time 0, 0.45 mL/min; 19 min, 0.45 mL/min; 19.5 min, 0.55 mL/min; 27 min, 0.55 mL/min; 27.5 min, 0.45 mL/min; and 28 min, 0.45 mL/min.

The table generated after processing the individual metabolites was used to carry out multivariate analysis of the data. For this, we used MetaboAnalyst 5.0 online software. Principal component analysis and partial least squares discriminant analysis (PLS-DA) were performed on the log-transformed data and standardized by Pareto scaling. Principal component analysis was used to observe grouping and sample discrepancies in general, while PLS-DA was used to maximize the variations and to guarantee the discriminatory effect of the components based on the values of the variable importance in projection (VIP).

A cross-validation test was applied to validate the method created by PLS-DA and indicated which component of the model was the one that best explained the variations in ions between the groups. Based on the PLS-DA, we selected the metabolites with the highest VIP scores from the component with the greatest discriminatory effect as potential biomarkers. We built an ROC curve for the metabolite combinations. In addition, we performed a permutation test (1000x) and calculated the prediction class probability based on our samples.

### Pathway analysis

Pathway analysis using MetaboAnalyst was conducted to identify the affected pathways based on the identified metabolites and their concentrations obtained from the PLS-DA. MetaboAnalyst 5.0 software was employed with the Homo sapiens (hsa—KEGG organisms) Pathway Library. This analysis compared the presence of metabolites with the pathways described in the hsa—KEGG database, considering the number of links each metabolite had within a pathway. The results provided potential affected pathways based on the identified metabolites in the study.

### Statistical analysis

The analysis of clinical data between the groups was performed using the non parametric 2-sided Mann‒Whitney U test, using SPSS (IBM SPSS Software version 22). The results obtained from the patients’ clinical data are expressed as the median (quartiles) or numbers (percentages). Furthermore, multivariate statistics were performed using MetaboAnalyst 5.0 software. Significant differences were considered when the *p* value was < 0.05. Graphs were created with MetaboAnalyst 5.0 software and the GraphPad Prism 8 program.

### Informed consent

Informed consent was obtained from all subjects involved in the study.

## Data Availability

The data obtained such as the concentration of each metabolite identified in the FF and its normalization are available in an online repository and can be found under the number: https://doi.org/10.17632/nm5gzmdsjh.1. at: https://data.mendeley.com/datasets/nm5gzmdsjh.
